# *Mycoplasma pneumoniae* Epidemiology in England and Wales: A National Perspective

**DOI:** 10.3389/fmicb.2016.00157

**Published:** 2016-02-16

**Authors:** Rebecca J. Brown, Patrick Nguipdop-Djomo, Hongxin Zhao, Elaine Stanford, O. Brad Spiller, Victoria J. Chalker

**Affiliations:** ^1^Public Health EnglandLondon, UK; ^2^Department of Child Health, University Hospital Wales, Cardiff University School of MedicineCardiff, UK; ^3^Faculty of Epidemiology and Population Health, London School of Hygiene and Tropical MedicineLondon, UK

**Keywords:** *Mycoplasma pneumoniae*, epidemiology, England, wales, microbiology

## Abstract

Investigations of patients with suspected *Mycoplasma pneumoniae* infection have been undertaken in England since the early 1970s. *M. pneumoniae* is a respiratory pathogen that is a common cause of pneumonia and may cause serious sequelae such as encephalitis and has been documented in children with persistent cough. The pathogen is found in all age groups, with higher prevalence in children aged 5–14 years. In England, recurrent epidemic periods have occurred at ~4-yearly intervals. In addition, low-level sporadic infection occurs with seasonal peaks from December to February. Voluntarily reports from regional laboratories and hospitals in England from 1975 to 2015 were collated by Public Health England for epidemiological analysis. Further data pertaining cases of note and specimens submitted to Public Health England from 2005 to 2015 for confirmation, molecular typing is included.

## Introduction

*Mycoplasma pneumoniae* is a respiratory bacterial pathogen causing upper and lower respiratory disease in humans of all ages. It is a major cause of community-acquired pneumonia (CAP) and is considered to be responsible for 15–20% of CAP cases in adults and up to 40% of cases in children, especially in children of school age (Foy, 1993; Korppi et al., 2004; Dumke et al., 2012). Up to 25% of *M. pneumoniae* infections may manifest as extra-pulmonary sequelae after the onset of or in some cases in the absence of respiratory illness (Cassell and Cole, 1981; Narita, 2010). Encephalitis is one of the most severe complications (Narita, 2009; Meyer Sauteur et al., 2014b) estimated in 5–10% of pediatric encephalitis patients (Bitnun et al., 2001; Christie et al., 2007) of which up to 60% of have additional neurologic sequelae (Bitnun et al., 2001, 2003). *M. pneumoniae* infection can result in dermatological manifestations including Stevens-Johnson syndrome (Olson et al., 2015). Hemolytic anemia is a rare but serious complication of *M. pneumoniae* infection and is more frequent children than in adults (Gu et al., 2014). *M. pneumoniae* infections occur both endemically and epidemically worldwide, with epidemic peaks every 4–7 years (Chalker et al., 2011a, 2012a; Jacobs, 2012). Typical outbreaks of *M. pneumoniae* infection occur in areas of close personal contact for example, schools and military barracks. Both symptomatic and asymptomatic individuals with *M. pneumoniae* carry the organism in the respiratory tract and it can be transmitted from person to person via aerosols and cough (Clyde, 1979; Waites and Talkington, 2004; Meyer Sauteur et al., 2014c). Long-term morbidity due to *M. pneumoniae* infection is uncommon however; the acute illness is often disruptive and can consume significant resources (Waites and Talkington, 2004).

In England and Wales (EW), seasonal peaks of infection are detected from December to February each year with epidemics at ~4-yearly intervals (Chalker et al., 2011b, 2015). Cyclical patterns, as observed in EW, are also seen in Denmark, Sweden, Norway, Finland, Korea, and Japan (Ito et al., 2001; Rasmussen et al., 2010; Blystad et al., 2012; Linde et al., 2012; Polkowska et al., 2012; Kim et al., 2015). It has recently been suggested that minor variations in the duration of immunity may be essential to the cyclic epidemic peaks (Omori et al., 2015). A con-current increase in reported *M. pneumoniae* cases was documented in several European countries in 2011 (Lenglet et al., 2012) and in EW the most recent increase has been noted in 2015 (this study).

The recent global increase in macrolide resistance observed in cases of *M. pneumoniae* infection is of increasing concern and importance to the international community (Bébéar, 2012). In China resistance has been documents in over 90% of clinical isolates of *M. pneumoniae* studied (Zhao et al., 2013) however resistance is lower in European counties including France, Germany, Switzerland, and Sweden (Peuchant et al., 2009; Meyer Sauteur et al., 2014a; Nilsson et al., 2014; Dumke et al., 2015). Macrolides are currently recommended as the first-line treatment for *M. pneumoniae* infection in the UK (Harris et al., 2011). The 2011 British Thoracic Society guidelines for the management of CAP in children and adults suggest empirical macrolide treatment at any age if there is no response to first-line β-lactam antibiotics (which are ineffective against cell wall-less bacteria such as *M. pneumoniae*) or in the case of very severe disease (Lim et al., 2009; Harris et al., 2011). Macrolide-resistance in EW has recently been documented at 9.3% and is therefore not included in this article (Brown et al., 2015a). This is considerably lower than in Scotland (19%) (Ferguson et al., 2013).

This study aims to provide up to date overview of the number of laboratory reports and incidence of *M. pneumoniae* infection in EW, molecular typing data, and briefly highlight cases of note in recent years.

## Materials and methods

**Figure 1:**
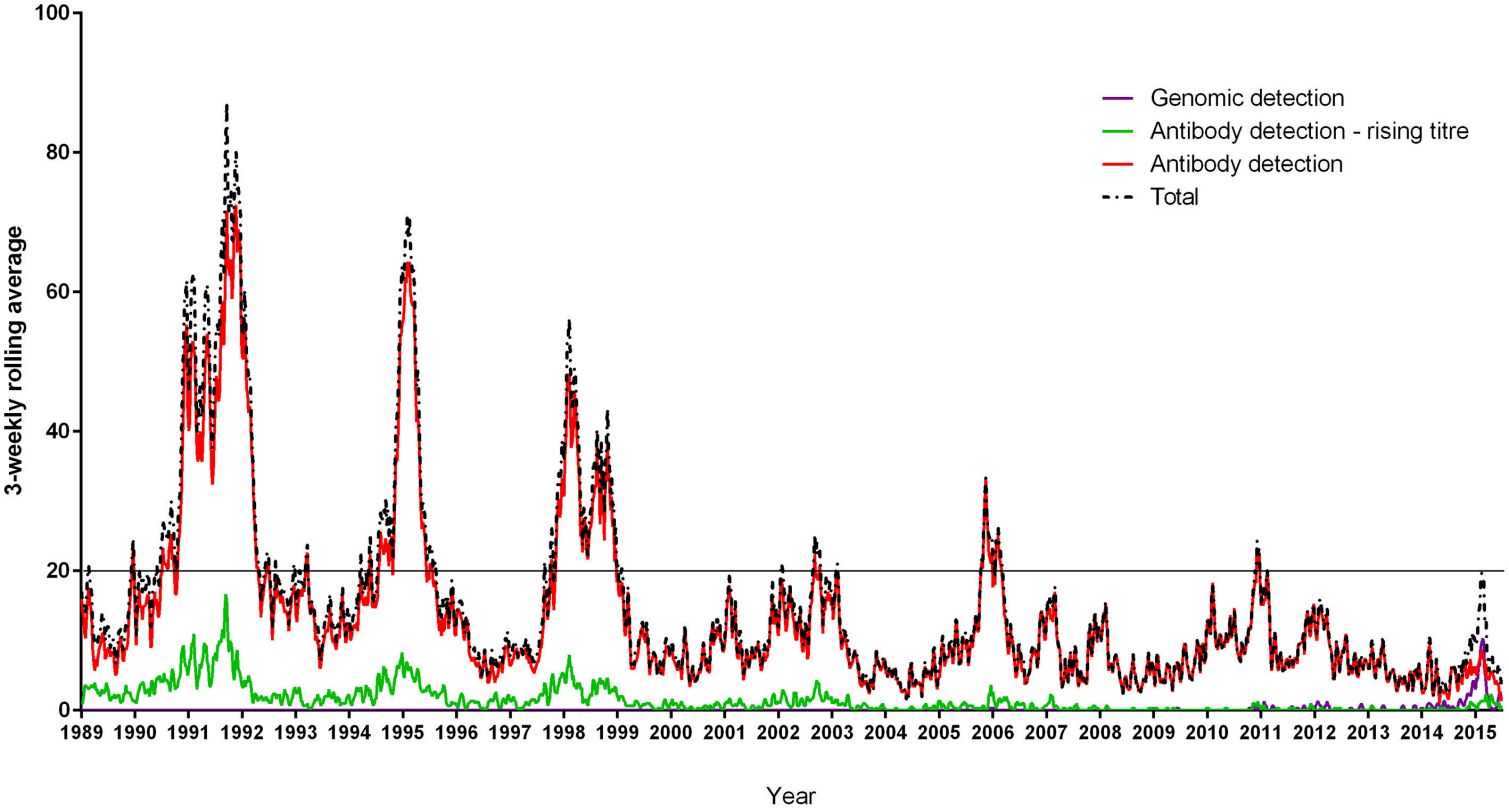
**Laboratory reports of *Mycoplasma pneumoniae* infection detection by genomic and serological methods in England and Wales from January 1989 to June 2015**. The line at 20 cases per 3 weekly average rolling period defines seven epidemic periods of declining magnitude and clarity, lasting up to 2 years (1991–1992, 1994–1995, 1998–1999, 2001–2003, 2005–2006, 2011, 2015). National reporting categories include antibody detection and antibody-detection rising titre. A rising titre is defined as a four-fold increase in detectable anti-*Mycoplasma pneumoniae* antibody level.

A total of 16,878 serological, culture, genomic, and unspecified laboratory diagnostic methodology *M. pneumoniae* positive cases reported to Public Health England, via the Communicable Disease Report Network comprising ~250 laboratories from January 1989 to June 2015 were aggregated into 3 weekly periods. These report the organisms identified from specimens (e.g., throat swabs, serum, or sputum) submitted by general practitioners and hospitals with the patient's age and sex, the reporting laboratory, and date of the first sample; the system has changed little over time. Duplicate specimens were removed and reports plotted to examine the general pattern (3-weekly moving average). National reporting categories include antibody detection and antibody-detection rising titre. A rising titre is defined as a four-fold increase in detectable anti-*M. pneumoniae* antibody level. Rising titres are not demonstrated for all patients as it is not always possible to obtain a second specimen. It is possible that a fraction of cases reported as antibody detection only include some cases of rising titre that have not been appropriately coded. A distinction between IgA, IgG and IgM cannot be made when collating figures, however the number of cases with rising titre demonstrating active infection mirror the overall total case pattern of epidemic periodicity. A total of 39,758 laboratory reports of *M. pneumoniae* infections in England and Wales from January 1975 to June 2009 previously examined indicated that cyclic epidemics occurred every 4 years, were synchronous across all regions in the country, and occurred during the winter (Nguipdop-Djomo et al., 2013). Epidemic periods were defined as a clear increase in cases resulting in more than 20 cases in a 3 weekly average rolling period (Figure 1). From this dataset, we computed average age specific incidence for epidemic and non-epidemic periods using the England and Wales (EW) population censuses of 1981, and 2001 for the denominator for the periods 1975–1988 and 1998–2009 respectively. Data from 1989 to 1997 were excluded from age-specific analyses because age was missing in ~90% records during that period. Age distribution incidence rates from 2010 to June 2015 were calculated using the Office for National Statistics (ONS) mid-year population estimates for EW.

**Figure 2:**
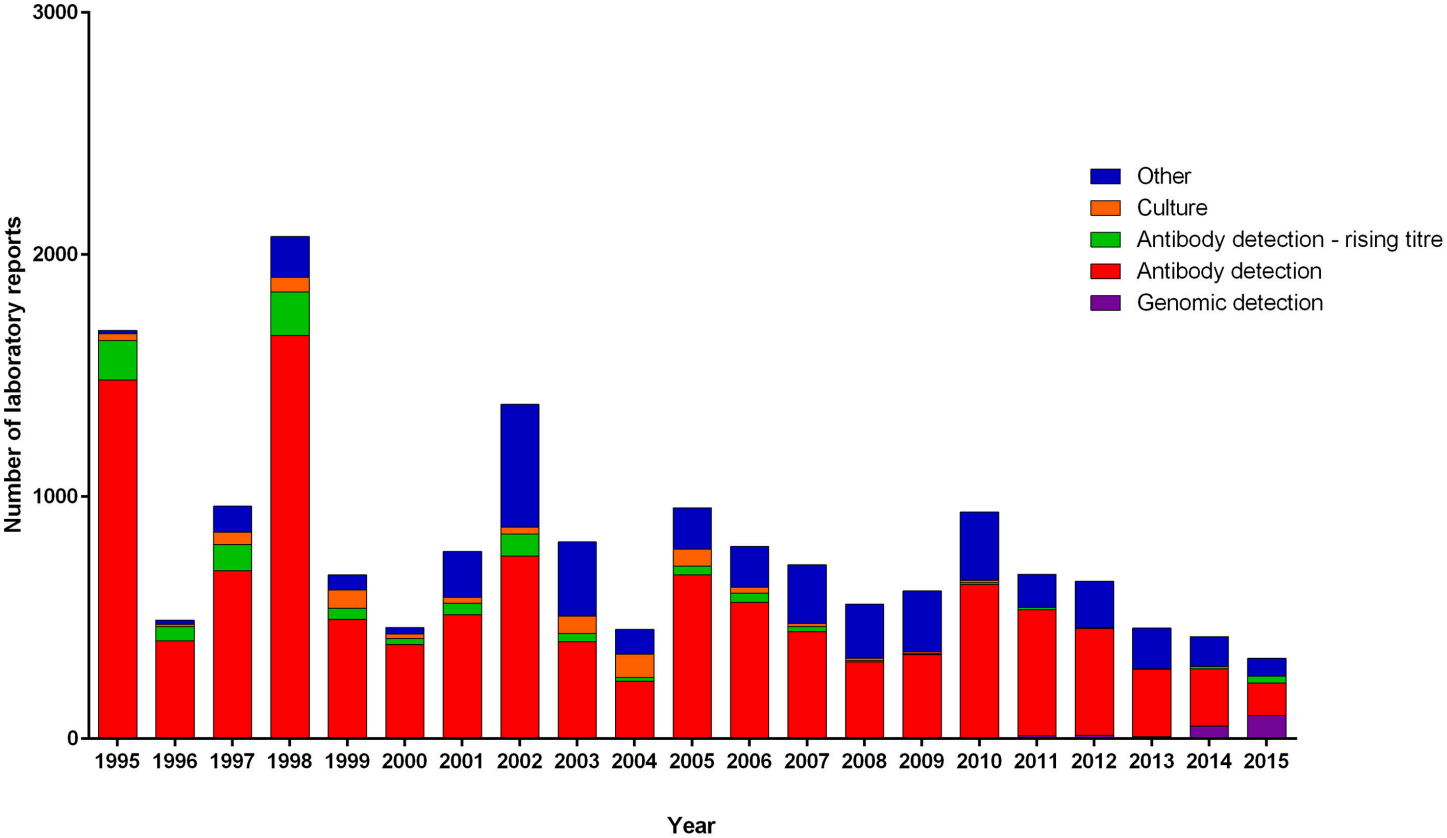
**Number of laboratory reports per year from January 1995 to June 2015 separated by detection methodology**. National reporting categories included are: antibody detection and antibody-detection rising titre. A rising titre is defined as a four-fold increase in detectable anti-*Mycoplasma pneumoniae* antibody level (methods not specified). Other indicates specimens for which *M. pneumoniae* infection was determined using antigen detection (method not specified), microscopy and unknown categories. Culture indicates cases from which specimens yielded isolates of *M. pneumoniae* and genomic detection those for which DNA of *M. pneumoniae* was detected by PCR.

**Figure 3:**
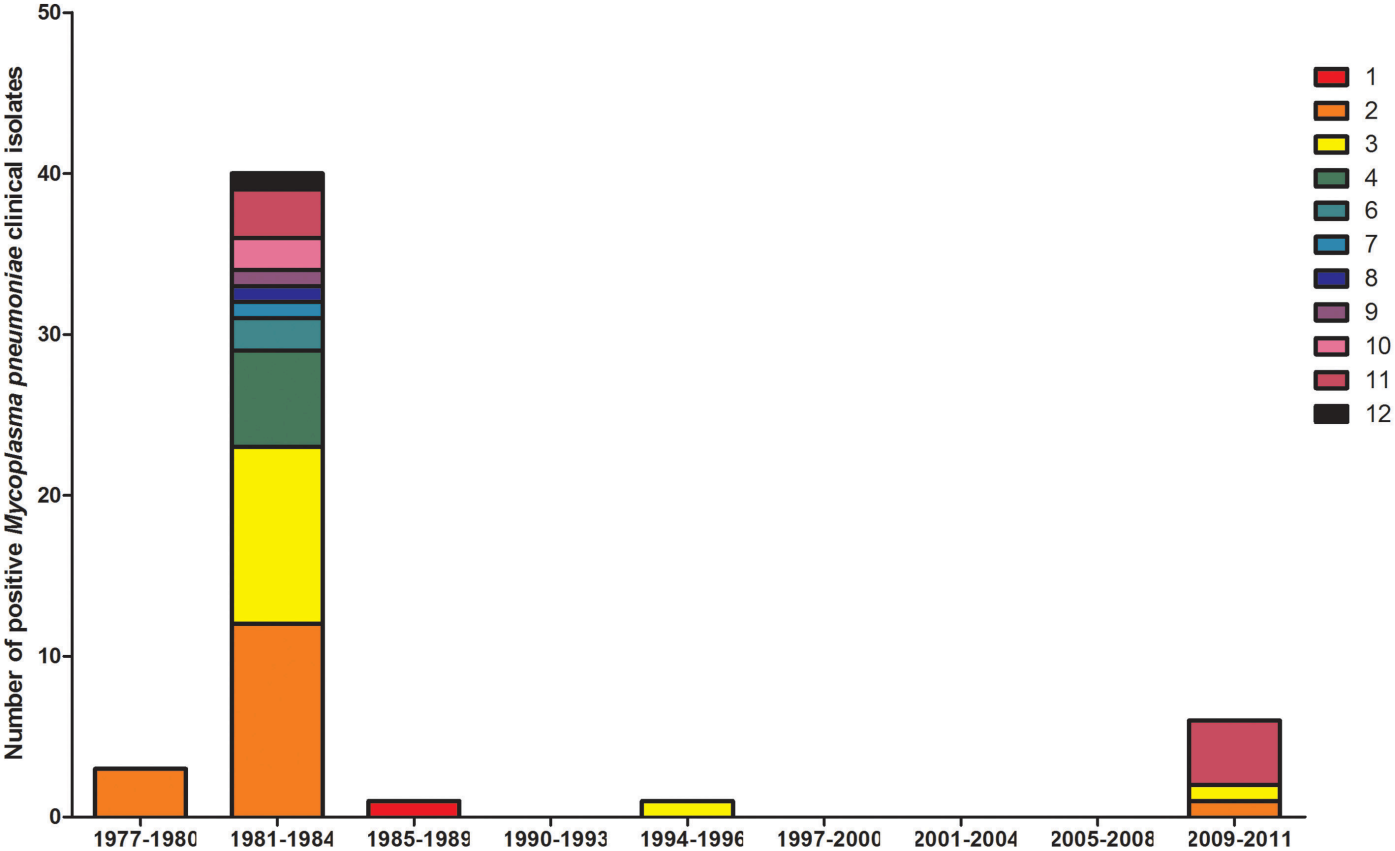
**Distribution of MLST sequence types for 57 *M. pneumoniae* clinical isolates in the 4-yearly epidemic cycles observed in the UK**. Year groups indicative of epidemic periods are listed on the *x*-axis. Sequence types (ST) 1–12 are listed in the key and indicated with differing colors. Allelic profiles are available on http://pubmlst.org/mpneumoniae.

**Figure 4:**
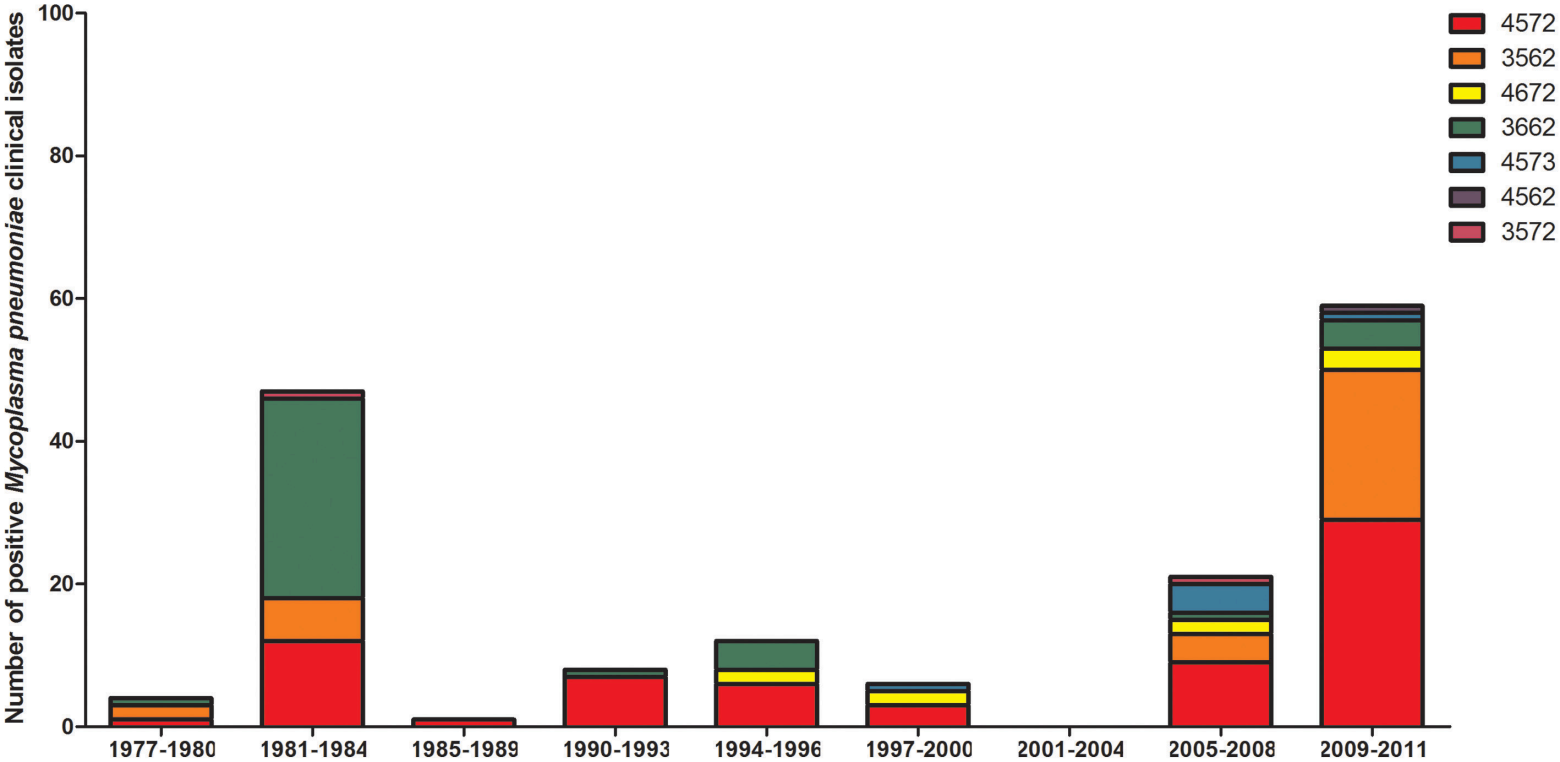
**Distribution of MLVA types in positive *M. pneumoniae* clinical specimens/isolates collated into the 4-yearly epidemic cycles observed in the UK**. Year groups indicative of epidemic periods are listed on the *x*-axis. MLVA profiles are listed in the key and indicated with differing colors.

**Figure 5:**
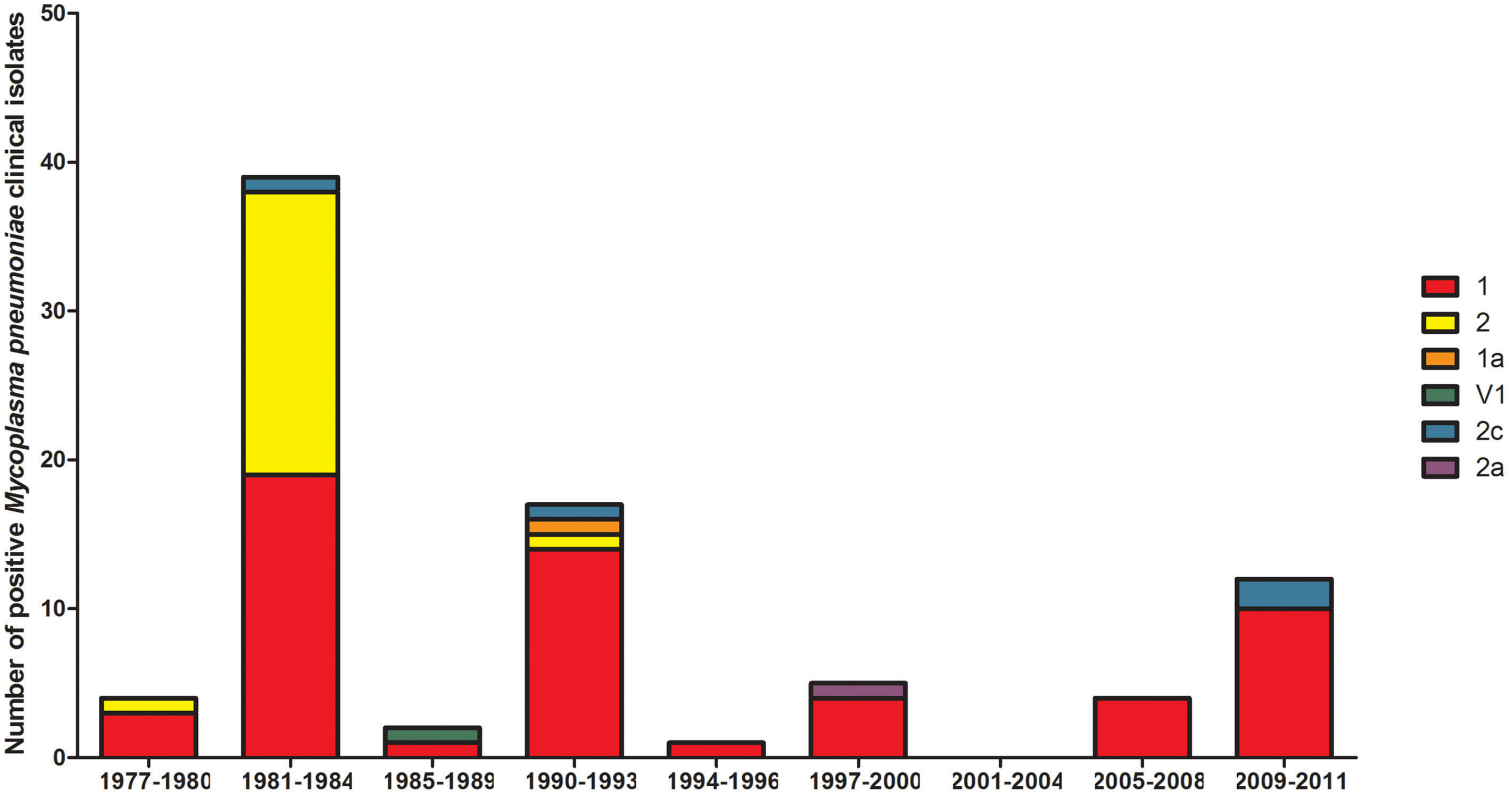
**Distribution of P1 type in 4-yearly epidemic cycles observed in the UK for positive *M. pneumoniae* clinical specimens/isolates**. Year groups indicative of epidemic periods are listed on the *x*-axis. P1 types are listed in the key and indicated with differing colors.

Diagnostic methodology of choice has altered with time in EW, with the decline in culture and use of the complement fixation test being superseded by enzyme immunosorbent assays and the introduction of molecular testing. To ascertain the proportion of reports now obtained using molecular methods, differing methodologies in use with time was examined from 1989 to 2015 (Figure 2). Molecular typing of *M. pneumoniae* positive clinical specimens and isolates was undertaken using MLST (50) (Brown et al., 2015b), MLVA (156) (Chalker et al., 2015), and P1 type (84) determinations (Dumke et al., 2006) from 1977 to 2011 (Figures 3–5). Referred cases to the Bacteriology Reference Department, Public Health England from 2005 to 2015 were examined for cases with unusual or severe presentation.

## Results

From January 1989 to June 2015 seven epidemics of *M. pneumoniae* were noted of declining amplitude with recent peak in 2015 (Figure 1). For some epidemic periods clear annual fluctuations can also be seen apparent as a double peak over two winter seasons. The clarity of epidemic periods have in recent years declined with less reported cases overall. From 1975 to 2009 incidence was found to be similar by gender, both during epidemic and inter-epidemic periods. The annual notification rate in 2010–2015 was consistently highest in those aged 15–44 years, detailed in Table 1. The use of culture has declined in recent years and despite serology being the most commonly used methodology the implementation and increased use of molecular methods has resulted in a proportional increase in reports based on molecular tests from 0.32% (3/936 95%CI 0.06–0.98) in 2010 to 28.5% (95/333 95%CI 24.0–33.6) in the first 6 months of 2015 (Figure 2). Molecular typing data were grouped into 4-yearly intervals, representing the epidemic cycles observed in the UK. Multiple MLST, MLVA, and P1 types were observed in each 4-yearly interval (3–5) however a predominance of P1 type 1 can be seen for all intervals except 1981–1984 where equal numbers of P1 type 1 and type 2 strains were observed. This data is limited by low sample number in 4-yearly intervals, therefore the variation in P1 types observed is likely to be an underestimate of the actual *M. pneumoniae* population present.

**Table 1:** **Incidence of *M. pneumoniae* positive laboratory reports per million persons**.

	**Year**	**Incidence (per million persons in overall population)**								
		**0–4 years**	**5–9 years**	**10–14 years**	**15–24 years**	**25–44 years**	**45–64 years**	**65+ years**	**Unknown**	**Total**
Serology + PCR	2010	1.17	1.35	0.99	1.40	2.09	1.44	0.61	0.00	9.05
	2011	1.14	1.83	1.16	1.51	2.40	1.28	0.87	0.02	10.21
	2012	1.45	1.47	1.15	1.45	2.84	2.05	1.22	0.00	11.63
	2013	0.61	0.95	0.74	0.93	1.77	1.82	1.42	0.00	8.25
	2014	0.82	0.78	0.47	0.89	2.09	1.10	1.31	0.02	7.47
	2015^*^	1.78	1.22	0.70	1.15	3.07	1.88	1.32	0.00	11.11
	Average	1.16	1.27	0.87	1.22	2.38	1.60	1.13	0.01	9.62

* Data for 2015 from January to June and rates adjusted for half-year data were collected.

### Cases of note: 2005–2015

From January 2005 to June 2015 eleven cases were referred to the Bacterial Reference Department, Public Health England that were identified as positive for *M. pneumoniae* that were of particular note. The majority of cases were patients with lower respiratory tract infection. Stevens-Johnson syndrome is an immune-mediated hypersensitivity complex typically involving the skin and mucous membranes. Two cases of Stevens-Johnsons syndrome were noted in 2009 and 2010 in male children aged 8 and 6 respectively. Two cases were noted in respiratory specimens in immunocompromised patients following extra-pulmonary organ transplantation (2013 and 2015). Infection in donor transplant patient respiratory secretions was also noted in 2015. *M. pneumoniae* was detected by qPCR in the nasopharyngeal aspirate but not the cerebral spinal fluid (CSF) of a patient with pneumonia and reactive transverse myelitis in a child in 2005, and in the bronchoalveolar lavage of a child with encephalitis and seizures in 2011. In 2011 a young adult patient presented post respiratory tract infection with encephalitis and transverse myelitis that progressed to tetraplegia with ventilator dependency. *M. pneumoniae* was confirmed by qPCR on throat swab specimens taken 19 and 21 days post onset but was not detected in concurrent CSF specimens (Chalker et al., 2012b). Detection of *M. pneumoniae* in CSF is unusual and it is postulated that neurological manifestation of *M. pneumoniae* infection is antibody mediated rather than by direct presence of the bacteria itself (Waites and Talkington, 2004). Of 68 CSF specimens referred only 1 positive case was detected in 2010, in a child with a ventriculoperitoneal shunt, in which contamination of the CSF during sampling could not be excluded. In 2012 *M. pneumoniae* was detected by qPCR in the lung tissue of *two* co-habiting adults that both suffered sudden fatal collapse. This was presumed a secondary infection as one of the two patients also had confirmed *Staphylococcus aureus* infection.

## Discussion

Laboratory reports show that cyclic epidemics of *M. pneumoniae* infections in EW recur every 4 years on average, concurrent with annual seasonal fluctuations with incidence peaking and dipping in the winter and summer respectively. The reduction in clarity and magnitude of epidemic periods in recent years could be indicative of a genuine reduction in cases, increasing population pulmonary health, or reflect the changing nature of testing strategies moving away from techniques such as complement fixation. Overall incidence, although declining over the period 1989–2015, has remained static since 2010 and age-specific differences in epidemic period incidence were noted for the limited periods studied. Annual notification rate in 2010–2015 was highest in 15–44 year olds perhaps reflecting reliance on serological confirmation or infection. Globally, epidemics of *M. pneumoniae* are considered to occur every 3–7 years, however recent epidemiological studies have documented varying trends in epidemic patterns. Serological studies performed in Denmark showed a pattern of *M. pneumoniae* infections over a 50 year period from 1946 through 1995 with endemic disease transmission punctuated with cyclic epidemics every 3–5 years (Lind et al., 1997). In Jerusalem, historically, epidemics were observed every 3–5 years with seasonal peaks in October and early spring; however, since autumn 2014 a constant rate of infection has been observed, diverging from the historical pattern (Nir-Paz et al., 2012). Indeed, similar to the data for EW; 3-yearly cyclic epidemic periods with declining magnitude have been documented in Japan from 1979 to 1999 (Ito et al., 2001).

Speculations regarding the mechanisms driving fluctuations in population incidence of *M. pneumoniae* infections have included decline in immunity or increase of the immunologically naive population level (Chalker et al., 2011a) or shifts in the proportion of individual strains with specific P1 type or concurrent increased incidence of several strains. Additionally, it is believed that the genotype of *M. pneumoniae* may be changing, generating diverse genetic material in each epidemic with a study reporting the detection of polyclonal strains in a single epidemic (Pereyre et al., 2012). Recent modeling of epidemic peaks has suggested that fluctuations may be attributable to minor variations in the duration of immunity at the population level (Omori et al., 2015). Speculation that a shift in P1 adhesin type may be the cause of epidemics has been disputed with evidence indicating the presence of multiple P1 adhesin types in observed increases of infection (Sasaki et al., 1996; Dégrange et al., 2009; Pereyre et al., 2012). It was hypothesized that a decline in immunity or an increase of the immunologically naïve population may result in the 4-year cycle of epidemic periods (Chalker et al., 2011a). In other geographical locations, it has also been observed that multiple P1 types can be detected during outbreaks, and it has been suggested that although immunological pressure may favor shifts of P1 type, a co-circulation of P1 types appears to be common (Nilsson et al., 2010; Dumke et al., 2015). This is further supported by the presence of multiple MLST types within specimens in EW, reflecting the concurrent presence of strains of varied genetic lineage. As expected an increase in molecular detection of infection is noted, with declining use of culture. Macrolide resistance has recently been documented at 9.3% found in adult patients only (Brown et al., 2015a) and is also of concern in children in other countries (Meyer Sauteur et al., 2014a). However, this was derived from results of specimens submitted to the reference laboratory which may be biased toward those developing resistance during treatment and one patient was documented to have received macrolides prior to sampling therefore this level may be an over-representation to the actual level in the community.

Extrapulmonary complications of *M. pneumoniae* infection can arise involving the skin and the nervous, cardiovascular, renal, gastrointestinal, musculoskeletal, and hematologic systems. The presence of *M. pneumoniae* in these extrapulmonary sites has been confirmed by PCR as well as culture (Koletsky and Weinstein, 1980; Kasahara et al., 1985; Narita et al., 1996; Saïd et al., 1999; Bar Meir et al., 2000). Complications that occur within the central nervous system (CNS) are recognized as the most common extrapulmonary manifestations of *M. pneumoniae* infections. A recent study of 1988 children with encephalitis showed *M. pneumoniae* as the most common causative agent (Bebear and Robertson, 1996). It is thought that the host immune response that develops after *M. pneumoniae* infection contributes to these complications as well as contributing to autoimmunity (Waites and Talkington, 2004). The mechanisms that result in these neurological manifestations of *M. pneumoniae* infection are not completely understood however, immune-mediated mechanism are suspected due to the development of cross-reactive antibodies to the brain and other neurological structures (Waites et al., 2008). PCR testing of 68 CSF specimens in EW resulted in detection of *M. pneumoniae* DNA in a single specimen in which contamination during sampling could not be excluded. Consideration to testing of CSF specimens in tandem with paired respiratory specimens should be given as in practice detection of *M. pneumoniae* in CSF is extremely rare and positive patients may be detected using pulmonary specimens.

In summary, epidemics of *M pneumoniae* infections recur every 4 years on average in EW affecting all age groups, predominantly children and adults <44 years of age. Macrolide resistance has recently been documented at 9.3% and extrapulmonary complications can be severe.

## Author contributions

RB worte the manuscript, PN, HZ, and ES undertook epidemiological data, OS and VC oversaw the study and wrote the manuscript.

## Funding

RB studentship was funded by Cardiff University and Public Health England, all other works was funded by Public Health England.

### Conflict of interest statement

The authors declare that the research was conducted in the absence of any commercial or financial relationships that could be construed as a potential conflict of interest.
